# Density of invasive western honey bee (*Apis mellifera*) colonies in fragmented woodlands indicates potential for large impacts on native species

**DOI:** 10.1038/s41598-022-07635-0

**Published:** 2022-03-04

**Authors:** Saul A. Cunningham, Mason J. Crane, Maldwyn J. Evans, Kassel L. Hingee, David B. Lindenmayer

**Affiliations:** 1 Fenner School of Environment and Society, The Australian National University, Canberra, ACT 0200 Australia; 2 Department of Ecosystem Studies, Graduate School of Agricultural and Life Sciences, The University of Tokyo, Tokyo, Japan

**Keywords:** Conservation biology, Invasive species

## Abstract

Feral *Apis mellifera* colonies are widespread globally and cause ecological impacts as pollinators and competitors for food and nesting opportunities. The magnitude of impact depends on their population density, but knowledge of this density is poor. We document feral *A. mellifera* colonies at 69 per km^2^ in fragmented *Eucalyptus* woodlands in Australia, exceeding estimates from elsewhere in the world, and matched only by one other Australian study. We surveyed 52.5 ha of woodland patches with 357 nest boxes installed to provide nesting opportunities for threatened vertebrates. Our sites covered a region of more than 140 km across with repeated surveys over 3 to 6 years. We show that nest box use by feral *A. mellifera* colonies is influenced by box design (p = 0.042), with weak evidence for an interactive effect of type of vegetation at a site (woodland remnants vs. replanting) and woody cover within 500 m (p = 0.091). At 69 colonies per km^2^, this density is equivalent to the recommended stocking of hives for pollination of some crops and is therefore likely to influence pollination and lead to competition with other flower visitors. *Apis mellifera* is also likely to be competing for hollows with cavity dependent native fauna, especially in landscapes where there has been extensive tree removal.

## Introduction

The Western honey bee (*Apis mellifera*) has been managed widely around the world, causing large ecological impacts as a pollinator and as a competitor for nesting and food resources^1^. *Apis mellifera* has also escaped from domestication to establish feral populations in many countries^2–4^, where it can have negative impacts on native biodiversity^5–8^. These feral bees also play a major role in pollinating economically significant crops^9^ and native plants^4^. The abundance of feral *A. mellifera* colonies in wild settings is thought to have declined in many regions worldwide, particularly as the parasitic mite *Varroa destructor* spread from Asia to the rest of the world^10,11^. At the same time, Africanized honey bees (i.e., those of European descent that have hybridized with the subspecies *A. m. scutellata*) have spread in the Americas and shown various levels of tolerance to *Varroa*^12^, so that a new wave of invasion has brought new impacts on pollinator networks and pollination outcomes^13,14^. However, the spatial distribution of feral *A. mellifera* colonies is poorly understood because data are difficult to collect.

As eusocial insects, the Western honey bee has been recognized as a particularly impactful invasive species because it can rapidly increase population in its invasive range and exploit resources (such as nectar and pollen) with a coordinated cohort of foragers that exceeds what can be achieved by non-social insects in the same region^15^. While the ecological impact of feral *A. mellifera* can reasonably be expected to relate to colony density^16^, there is no established baseline for determining when impacts of relevance to conservation management might be expected to occur. We can, however, compare the recommended “hive stocking rate” density for managed hives used for crop pollination to the density of feral colonies. A density high enough to ensure pollination of a monoculture crop can be assumed to be high in terms of the bees’ interactions with flowers of native vegetation. Hive stocking rates vary greatly across different crop types, but a recent review established that recommended rates range from around 50 up to greater than 1000 colonies per km^2^^17^.

Feral *A. mellifera* bees depend primarily on tree hollows to house their colonies, although artificial structures such as wall cavities in buildings or water meter boxes are also used where opportunities exist [e.g., Ref.^18^]. Direct feral colony surveys are labour intensive because this species can live in cavities high above the ground and scattered widely across the landscape. As a result, few studies report patterns from large area surveys. Ratnieks et al*.*^19^ assembled data from nine studies across the world and reported densities ranging from 0.17 to 9 colonies per km^2^. Subsequently Baum et al*.*^20^ recorded a density of 12.5 colonies per km^2^ in coastal prairie in Texas, USA, though a similar survey 13 years later found that the density had declined to 5.4 colonies per km^2^^21^. Oldroyd et al*.*^22^ surveyed 35 ha of woodland in Victoria, Australia and reported densities that varied between years from 50 to 150 colonies per km^2^. The density reported in Oldroyd et al*.*^22^ has been described as the highest ever recorded, placing it in the range of hive stocking rates for crop pollination, and was therefore characterized as atypical^23^. However, given the shortage of similar studies worldwide, the basis for cross-study comparisons is poor.

In common with other hollow-dependent fauna, their reliance on tree hollows means that feral *A. mellifera* populations can be reduced by tree clearing, and subsequent recovery can be slow because development of hollows is slow and typically occurs in old trees^24^. Where there are multiple species competing for limited tree cavities, it is also possible that successful invaders, such as *A. mellifera*, can displace native fauna^25^. For this reason, competition with feral honey bees is listed as a key threatening process for endangered tree cavity-dwelling species in environmental protection legislation in the Australian state of New South Wales^26^. One of the strategies commonly employed to recover populations of declining hollow-dependent fauna is to install artificial nest boxes^27^, but if these boxes are occupied by honey bees, then the strategy can be undermined. Honey bees have been reported as users of nest boxes in many landscapes^28–30^ and in some cases are reported as among the most frequent users of these artificial structures^31,32^.

Assessing artificial nest box use by *A. mellifera* is more tractable than surveying natural hollows because the location of the nest boxes is known and can be controlled. However, studies of artificial nest boxes commonly report results only in terms of the proportion of nest boxes occupied [e.g., Ref.^31^]. While this gives insight into potential competition for nesting opportunities, it does not allow the calculation of the landscape density of honey bee colonies, which is particularly important for understanding the ecosystem-scale impacts of this species.

Another method that is developing for estimating the density of *A. mellifera* colonies at large scales is trapping males (drones) with pheromone-baited lures and then using genetic methods to discriminate different brother groups^33,34^. This approach requires a number of assumptions to be validated, including the flight distance for drones and the proportion of colonies contributing drones to the sample. Utaipanon et al*.*^35^ showed that drones can fly up to 3.75 km to a lure, but there were insufficient data (only two transects) to estimate confidence intervals around this estimate. Further, recent field trials showed that only 64% of known colonies within a 1.55-km in radius contributed drones to the trapped sample, with fewer drones being contributed by hives that were further away^36^. In addition, even at large sample sizes, the average number of drones contributed per colony was lower than the number required to identify brother groups accurately^36^. As a consequence, estimated colony densities based on drone trapping are difficult to interpret and likely are substantially underestimating the true colony density in a given area, therefore invalidating direct comparisons between densities established by ground survey data and densities reported by drone trapping studies [e.g., Ref.^21^].

In this study we analysed the use of artificial nest boxes by *A. mellifera* colonies in a region of southern New South Wales, Australia, and ask if the density of invasive feral bee colonies in this landscape is high enough to reasonably expect there to be important impacts on native biodiversity. By combining survey data from several different studies in the same region, we compiled a large data set with 566 nest boxes, each surveyed at least three times over four years. The data were refined to a subset of 357 nest boxes used to estimate the density of colonies, and a different subset of 299 nest boxes used to model the probability of occupancy, including vegetation structure (by comparing remnant woodland and re-planted woodland), woodland cover at a landscape scale, and nest box design.

The use of artificial nest boxes by feral honey bee colonies establishes a lower bound for the true density of colonies in this landscape, because uncounted colonies may also occur in natural hollows or in other structures. As we examined the use of recently established nest boxes, this study provides an indication of whether nesting opportunities strongly limit the density of feral *A. mellifera* colonies in this area. A high rate of box use by bees suggests that new nesting opportunities provided by artificial nest boxes allowed for an expansion of the pre-existing population. If nest box use is rare, then this would indicate either that the established bee population (i.e., the source for new colonies) is not expanding, or that there are so many better nesting opportunities available that artificial nest boxes are not as attractive as the natural resources available.

## Methods

### Field sites and surveys

**Figure 1:**
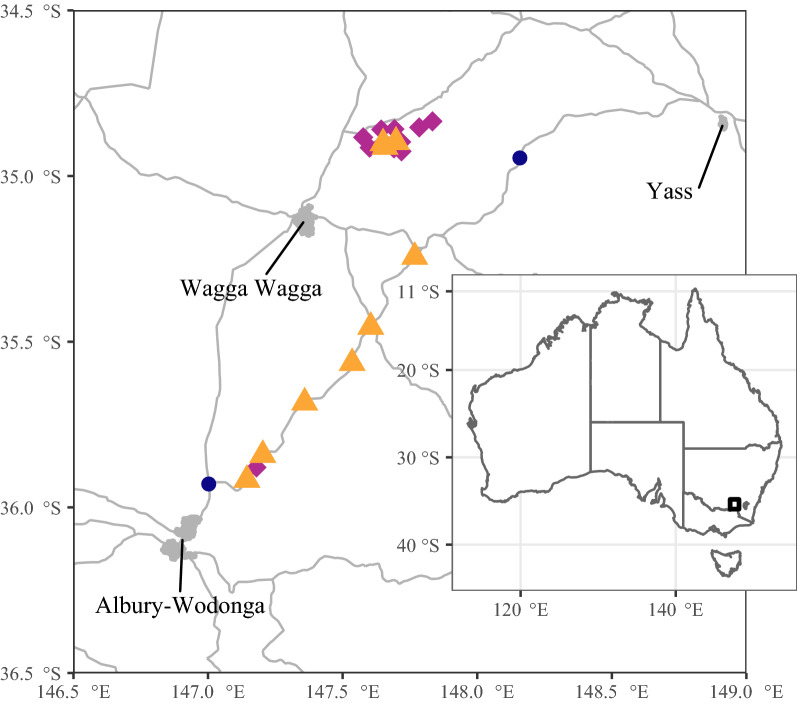
Map of the location of nest box survey sites. Triangles indicate sites that were used for calculating areal density of *Apis mellifera* colonies and modelling determinants of occupancy. Diamonds indicate sites used for occupancy modelling but not colony density calculations. Circles indicate sites that were included in the overall survey count but were not included in the occupancy modelling or density analyses. Figure created using R^39^ with base map data from Geoscience Australia^71^.

Artificial nest boxes were attached to trees at 23 sites in SE NSW, and were located mostly within 10 km of the Hume Highway in a band extending from the towns of Albury in the southwest to Yass in the northeast, with an additional cluster of sites 40 km north of the highway (Fig. 1). The northernmost site was separated from the southernmost site by 148 km.

Wooden nest boxes of various designs were installed to accommodate the requirements of a wide range of different hollow-using fauna. Nest boxes were in place for at least eight months before the first survey. The first surveys for nest box occupation were conducted in July 2010 and the final surveys were conducted in November 2014. Repeat surveys were separated by at least four months. Sites contained between six and 72 nest boxes, with 566 boxes in total being surveyed at least three times and as many as six times over the survey period (average = 5.11 surveys per site).

During the surveys, boxes were visually inspected for current occupancy or evidence of use. This included noting the presence of live honey bee colonies or evidence that bees had used the box but were now absent, such as the presence of honeycomb. Other animals were noted also, but were not the focus of the current study. This work was conducted under animal ethics permits approved by the Australian National University and the New South Wales Office of Environment and Heritage.

### Landscape metrics

The dominant vegetation type across this landscape before clearing for agriculture was temperate *Eucalyptus* woodland^37^. Most of the boxes were placed in remnant woodland sites, left aside and fragmented when the rest of the landscape was cleared for agriculture. Some sites included woodland that had been created by replanting a formerly cleared area and therefore supported younger trees. We refer to these areas as “plantings” and placed boxes only in trees exceeding 6 m in height. Some sites were composed entirely of replanted trees and one large site was a complex mix of remnant and planted woodland.

To determine site area, we used a convex hull around the nest box locations to create polygons and added a 15-m buffer to maintain connection in the polygon across scattered woodland trees. This buffer was necessary because trees in these woodlands are widely spaced, even where there has been no land clearing. We then intersected the polygons with a 5-m resolution map of tree canopy based on SPOT5 imagery in the period 2008–2011^38^. This analysis was performed in R^39^.

Some of the sites were in roadside strips of vegetation and were therefore long and narrow, making calculations of a meaningful site area difficult. With the goal of calculating the areal density of bee colonies, we trimmed the data set to those sites with an area greater than 1 ha, and excluded the eleven smallest and narrowest sites. This approach left ten sites ranging from 2.4 to 12.8 ha, with an aggregate area of 52.5 ha containing 357 nest boxes.

For each nest box, we also calculated the amount of woody cover in the nearby landscape, using the same woody cover layer^38^. We calculated the proportion of a 100-m radius circle, centered on each nest box, that was occupied by woody cover, and then calculated the same proportion in a 500-m radius circle. As the center was always a tree (i.e., the tree with the nest box), the calculated proportions were always greater than zero.

### Statistical modelling

Sixteen different box designs were deployed across the whole study. For statistical modelling, we refined the data set to exclude nest box types for which there were fewer than 25 replicates. We also excluded one large site in which remnant and planted trees were interspersed because this structure was atypical in our design and in the broader landscape. The refined data set included 21 sites with a total of 299 nest boxes of four different types (Tables 1, 2). Five sites included areas of remnant and planting, ten sites were remnant only, and six sites were entirely composed of replanted trees (Table 2). The nest box types included medium sized boxes for smaller gliders and phascogales (*Petaurus breviceps*, *Phascogale tapoatafa,* type A) larger boxes designed for arboreal mammals such as squirrel gliders (*Petaurus norfolcensus*, type B), medium sized boxes for parrots (including the superb parrot, *Polytelis swainsonii*, type C), and smaller boxes for birds such as the brown treecreeper (*Climacteris picumnus*, type D).

**Table 1:** Attributes of the four different types of nest box analysed in the main analysis (dimensions in mm). All boxes were constructed of timber, with circular entry holes in the upper half, and attached to trees at 2.4–8.5 m height.

Type	Depth (mm)	Width (mm)	Height (mm)	Volume (litres)	Entrance diameter (mm)	Entrance area (mm^2^)
A	170	170	500	14.5	40	1257
B	300	300	500	45	80	5027
C	200	200	550	22	90	6362
D	150	150	150	3.4	50	1964

**Table 2:** Number of nest boxes by type (A, B, C, D), site (name given to a location), and site type (i.e., planting or remnant).

Site	Planting				Remnant				Total
	A	B	C	D	A	B	C	D	
BAL	4	2	2						8
BIMB	4	2	2						8
CP	3	2	2						7
LEH	4	1	2						7
MARY	4	2	2						8
SLI	4	2	2						8
BM						10	1		11
HOL					10	3	1	6	20
KYA					20	5	6	18	49
LB					10	1	6	10	27
NUN					4	2	2		8
ROSEV					4	2	2		8
RYA					4	4	3	2	13
SAG							1	9	10
SG					16				16
TAR					16	12	11	13	52
CREST	2	1	1		2	1	1		8
GLEN	2	1	1		2	1	1		8
GUN	2	1	1		2	1	1		8
HAZ	2	1	1		2	1	1		8
ROSEG	1	1	1		2	1	1		7
Total	32	16	17		94	44	38	58	299

To test which factors predicted the presence of honey bee colonies in nest boxes, we employed generalised linear modelling. Our response variable was the presence or absence of bee colonies in nest boxes in spring 2011. Our predictor variables were nest box type, site type, proportion of woody cover within 100 m of site, proportion of woody cover within 500 m of site, and site (Table 3).

**Table 3:** Variables used in the statistical modelling.

Variable	Type	Data	Details
Presence of honey bee colony	Response	Binomial	Presence (1), absence (0)
Nest box type	Predictor	Factor	Four types: A, B, C, D We used ‘D’ as the reference level as there were zero instances of bee colonies in this nest box type
Site type	Predictor	Factor	Nest boxes were located in either plantings or remnants
Proportion of woody cover within 100 m of site	Predictor	Continuous	Proportion between 0.03 and 0.99
Proportion of woody cover within 500 m of site	Predictor	Continuous	Proportion between 0.01 and 0.63
Site	Predictor	Factor	Site of nest boxes

We employed a model averaging procedure using Akaike’s Information Criterion corrected for small sample sizes (AICc^40^). We initially fitted a generalised linear mixed model (GLMM) containing all predictor variables and their interactions against the honey bee colony presence/absence variable, assuming a binomial error distribution. We included ‘site’ as a random effect to account for the possibility of inherent differences in suitability for honey bees among sites. After this preliminary fitting step, we determined that our model exhibited complete separation^41^. We therefore fitted subsequent models using a bias correction^42,43^, which is currently implemented only for generalised linear models (GLM). We included site as a fixed effect in our final models allowing this term to drop should it be considered unimportant to bee colony presence/absence during model selection. We fitted all subsets of the full model and performed model averaging on all models below ΔAICc = 6^40,44^. We used R for analyses and plotting^39^ using multiple packages^43,45–55^.

## Results

Feral *A. mellifera* colonies were widespread across the study region, with all but two (of 23) sites showing signs of use of nest boxes by colonies (Supplementary Information). Use by honey bees was recorded in 111 of the 566 boxes. In 26 cases, there was evidence of past use, but live colonies were not present at the time of the survey, so we did not include these records in the analysis. In 35 cases, nest boxes were occupied for a period of time but were abandoned before the final survey. The remaining 50 records were boxes that honey bees occupied and were still present on the final survey. The survey with the highest level of nest box use was recorded in spring 2011, during which we detected 36 occupied nest boxes across the 52.5-ha area covered by the 10 large sites, revealing an estimated density of 69 colonies per km^2^ of woodland. The proportion of nest boxes occupied by honey bee colonies was very similar in the survey conducted one year later, in spring 2012.

Two of the nest box types excluded from the refined data set hosted bees on at least some surveys. The first (similar to type B but with a 60-mm diameter hole) was excluded from further analysis because it was deployed only in one site (SAG) and had few records (eight). Five of these were occupied at least for one survey, including two instances that persisted over multiple surveys. The other box type (d = 170 mm, w = 170 mm, h = 500 mm, hole = 40 mm diameter) was deployed 24 times in two remnant sites, and live bees were detected in five different boxes across the three surveys but no hive persisted for more than one survey period.

Evidence of nest box use by honey bees was recorded in 90 out of 299 nest boxes of the refined data set. Of these, there were 45 records of boxes (15.1%) that bees occupied and were still present in the last survey, as well as 25 cases of boxes (8.3%) that were occupied for a period of time but were then abandoned. In 20 cases (6.6%) there was evidence of use by bees, but live bees were not present at the time of the survey, so we did not include them in the statistical analysis. Occupation by bees sometimes led to nest boxes being lost from future survey, with some boxes collapsing under the weight of the hive and others going missing in circumstances that suggest they were removed by people seeking to return bees to domestication.

**Figure 2:**
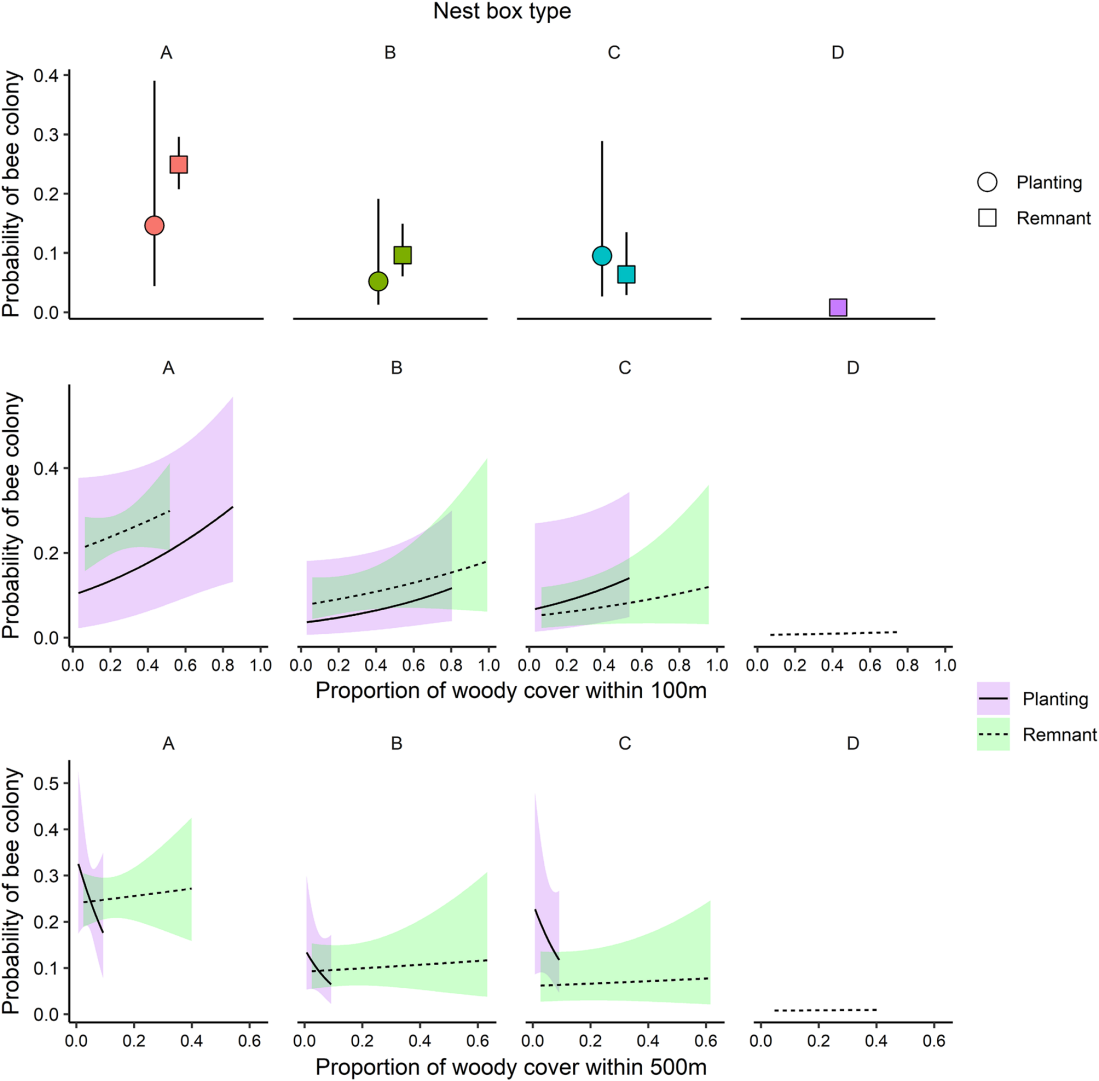
Model predictions for terms in the averaged model (AICc < 6). Errors represent standard errors around the mean (on the link scale). We plotted predictions for only those values within the range of the data. We omitted errors for nest box type ‘D’ from the plot because there were no honey bee colonies present in any of these nest boxes. Figure plotted using the ‘ggplot2’^52^ and ‘ggpubr’^47^ packages in R version 4.0.2^39^.

Focusing on the spring 2011 survey, nest box type A had the highest probability of being occupied by bees, and this effect was significant when comparing all remnant sites (p = 0.042; Table 4, Fig. 2). Confidence intervals were wider for the estimates from plantings, where there were fewer replicates. There was weak evidence for an interactive effect of site type and proportion of woody cover within 500 m (p = 0.091, Table 4), as colonies were more likely to be recorded in plantings than in remnants when the woody cover within 500 m was very low, whereas the pattern was reversed when the amount of woody cover increased. It is important to note that the maximum level of woody cover at 500 m was low for plantings but higher in remnants (Fig. 2). The other notable term in the averaged model was the interaction of woody cover within 100 m and site type (i.e., planting vs. remnant). Although the probability of next box use appeared to increase with woody cover at 100 m in both site types, neither of the terms on their own, or the interaction effect, were statistically significant.

**Table 4:** Model averaged coefficients (conditional) for all models below ΔAICc = 6. ‘~’ denotes that term is rank-deficient (i.e., there were no data for this term). We used nest box type ‘D’ and site type ‘planting’ as factor reference levels. *P* probability, *SE* standard error.

Model term	Estimate	SE	P
(Intercept)	− 3.710	2.058	0.072
Nest box type (A)	2.619	1.965	0.184
Nest box type (B)	1.481	2.046	0.470
Nest box type (C)	2.131	1.644	0.196
Proportion of woody cover within 100 m of site	2.506	1.891	0.186
Site type (remnant)	− 1.982	1.440	0.169
Nest box type (A): site type (remnant)	2.304	1.129	0.042*
Nest box type (B): site type (remnant)	2.305	1.441	0.111
Nest box type (C): site type (remnant)	~	~	~
Proportion of woody cover within 500 m of site	− 18.408	21.971	0.403
Site type (remnant): proportion of woody cover within 100 m of site	− 3.413	3.525	0.334
Site type (remnant): proportion of woody cover within 500 m of site	32.974	19.434	0.091

## Discussion

The widespread and rapid occupation of artificial nest boxes by feral *A. mellifera* colonies in this region of Australia indicates that there was an existing and widespread source population of honey bees previous to the start of the study that was sufficiently vigorous to support population growth in almost all the sites we surveyed. Possible sources for population expansion include both the pre-existing feral population and managed hives, which in this landscape are frequently placed near canola fields or near flowering *Eucalyptus melliodora*^56^, a common native tree in the region. The rapid occupation of nest boxes suggests that the availability of nectar and pollen in the landscape supports population growth, but suitable nesting opportunities are a limiting factor.

The density of feral honey bee colonies detected in this study (69 colonies per km^2^) is the lower bound for the true density because our data are only from artificial nest boxes. Nevertheless, even that level is very high compared to the other ground-based surveys of colony density, being 5.5 times higher than for Baum et al.^20^, the highest density recorded outside of Australia, and matched only by Oldroyd et al.^22^. Together, our study and Ref.^22^ suggest that such high densities of colonies may not be unusual in Australia. Given that our study spanned several years of repeated surveys (with similar densities found across 2 years) and covered sites from a wide region, our results cannot be dismissed as unrepresentative. It may be that Australia can support very high densities of feral honey bee colonies because of the abundance of nectar-rich native plant species and because the parasitic mite, *Varroa destructor*, has not yet gotten established in Australia^57,58^.

Studies using drone baiting and genetic analyses, including some from landscapes near to where we conducted this investigation^59^, have reported much lower estimated colony densities than we have. However, those studies should not be directly compared to our ground-based survey. In addition to known sources of underestimation (addressed in the “Introduction” section), those surveys defined the sampled area differently. Our study only included woody vegetation as a potential nesting habitat, and we calculated the area accordingly. In contrast, drone baiting studies sample from a landscape that includes potential nesting habitats as well as areas where there would be no suitable nesting opportunities for honey bees (e.g., fields cleared of all trees). Careful interpretation requires that the density concept being used is well matched to the question of interest. If the Baum et al.^20^ estimate (12.5 colonies per km^2^) is adjusted to reflect that only 56% of that landscape was woody vegetation of the kind likely to provide nesting opportunities to bees, the colony density could be re-expressed as 28 colonies per km^2^ of woody vegetation. Even at this higher level, the colony density we recorded in our study is 2.5 times higher than for the Baum et al.^20^ study.

### Is colony density high enough to impact the local ecosystem?

The woodland fragments that were the focus of our surveys occur in a broader landscape where woody vegetation has been extensively cleared and replaced with an agricultural system dominated by cropping and pastoralism^60^. While nesting opportunities are restricted to woodland fragments either in natural hollows or artificial nest boxes placed in trees, bees can forage over many kilometers^61^, and so the high density in the woodlands is supported by the collection of pollen and nectar over an area extending far beyond the bounds of small woodland patches. One could argue that the impacts of bees are dispersed (and therefore diluted) over a wider area than the woodlands that we focus on, and that our density estimate would be lower if it were calculated at that larger scale. We argue, however, that because native woodland fragments are hotspots of high biodiversity^62^, we should be concerned by the occurrence of such high colony densities in these patches because it is likely to bring local impacts even if the bee population uses resources collected from a wider area.

While many studies have established negative impacts of feral honey bees on local biodiversity^6^, there is no work connecting the scale of these impacts to the state of the broader honey bee population. The fact that we recorded values for colony density that exceeded all previous studies, bar one other from SE Australia^22^, gives us reason to be concerned that this population density would cause significant impacts to local species, including some that are vulnerable. However, the only approach available to give a comparative measure of the scale of impacts on floral resources and pollination is with recommended hive stocking rates in agriculture. At 69 colonies per km^2^, the local density exceeds some stocking rate recommendations for pollinating raspberry, vetch, and various melons and cucurbits^17^. Whereas the local density of honey bee colonies in our fragmented woodlands compares to these agricultural contexts, few native plants will produce flowers on a comparable scale to an agricultural crop, and the feral colonies maintain a year-round presence that is quite unlike the temporary provision of managed bees at peak flowering season in agriculture. Therefore, the abundance of bees relative to flower numbers in our sites can be expected to exceed levels in agriculture most of the time. This high abundance of bees relative to flowers can be expected to impact pollination rates of the many native plant species that attract *A. mellifera*^4^ and drive competition with native animals that rely on the same resources^7^.

The impact of abundant honey bee colonies exploiting flowers is an important dimension of biodiversity impact, but a different and additional set of species are impacted by competition for nests. It has already been established that the presence of *A. mellifera* discourages other species from using nest boxes in this region, particularly the vertebrate species that these nest boxes are intended to support^25^. Our analysis confirms the scale and extent of this problem. As we have focused on use of artificial nest boxes, our study provides no direct information regarding competition for natural hollows, but the rapid adoption of artificial nest boxes by honey bees is consistent with the understanding that natural cavities are in short supply.

Given the extensive use of tree hollows by *A. mellifera*, it is interesting to examine the value of scattered trees in heavily cleared landscapes for this invasive species. Our data suggest that the nest boxes placed in vegetation where there are unlikely to be natural hollows (i.e., plantings) are most likely to be occupied when there are few other trees (i.e., when woody cover is low) within 500 m. It may be that nesting opportunities are at a premium in landscapes that have crops that provide resources for honey bees (such as canola in our landscape^63^) but also are characterized by few trees that might support natural hollows. Mature paddock trees are known to provide natural hollows that are heavily in demand for other hollow-dependent fauna in these landscapes^64^ and are favoured by some vertebrate species^65^.

### Woody cover and box type affect occupancy

There are several ways in which vegetation structure (i.e., remnant woodland versus plantings) and woody cover might influence nest site selection by *A. mellifera*. For example, trees can provide both natural hollows for nesting (which might reduce use of nest boxes) and floral resources for bees (which might increase the number of colonies that can be supported). These complexities are particularly important in our study region where trees in the genus *Eucalyptus* are (a) widespread and dominant in treed landscapes, (b) provide highly valued food for honey bees^56^, and (c) known to provide many hollows^66^. It is not surprising, therefore, that these forest and landscape factors were important predictors of honey bees in our analysis and that the effects were complex and interacting.

The significant effect of box type on probability of occupation by bees is consistent with other studies that suggest an important role of nest box design^31^. The overall rate of nest box occupancy in our refined data set was 15.1%, but this rate includes one box type that was unused (type D) and another that had 23.8% occupation (type A). The preferred nest box design (type A) had a volume of 14.5 L, which places it in the observed range of natural cavities used by honey bees, though at the low end^67^. By contrast, at 3.4 L, nest box type D had a smaller volume than what is observed in natural cavities used by bees^67^, which may explain why it was not used by feral bees in our study. Seeley and Morse^67^ also report that nest openings in natural cavities chosen by honey bees had an area of 1000 mm^2^ to 4000 mm^2^, which indicates that the openings for box types B and C were larger than that normally preferred. These differences among nest boxes in suitability for *A. mellifera* need to be considered when comparing different studies. Innovation in nest box design might help to reduce the conflict between different users. Whereas all of the box types in our study were constructed of wood, a recent study showed that boxes made of PVC are less likely to attract honey bees and may therefore be more available to vertebrate fauna^32^.

## Conclusions

Our finding of high feral *A. mellifera* colony density has significant implications for conservation outcomes in this landscape, and indicates a similar risk for other landscapes where high colony density might develop. Impacts on pollination will be strong for many plant species, given that honey bees are the most common flower visitors in natural habitats globally, in both their native and invasive ranges^4^. Native bee communities are already known to be strongly influenced by ecological filters associated with fragmentation of habitat^68^, so the presence of an abundant invasive bee species will add another strong ecological filter. Many hollow-dependent animals that could compete with feral bee colonies for space might already be limited by the rarity of certain types of hollows^69^, and frequent use of artificial nest boxes by bees undermines the effectiveness of one of the most common conservation management interventions intended to moderate this problem. *Apis mellifera* is exceptional in most bee communities because of its large colony size. This is especially true in our study region, where there are no other eusocial bee species^70^. Further research is required to determine whether the high feral honey bee colony density documented in this study might also be found in the many other regions globally known to have feral honey bee populations, but for which no survey data are available to date.

## Supplementary Information

Supplementary Information.

## Data Availability

All data generated or analysed during this study are included in this published article and its supplementary information files.
